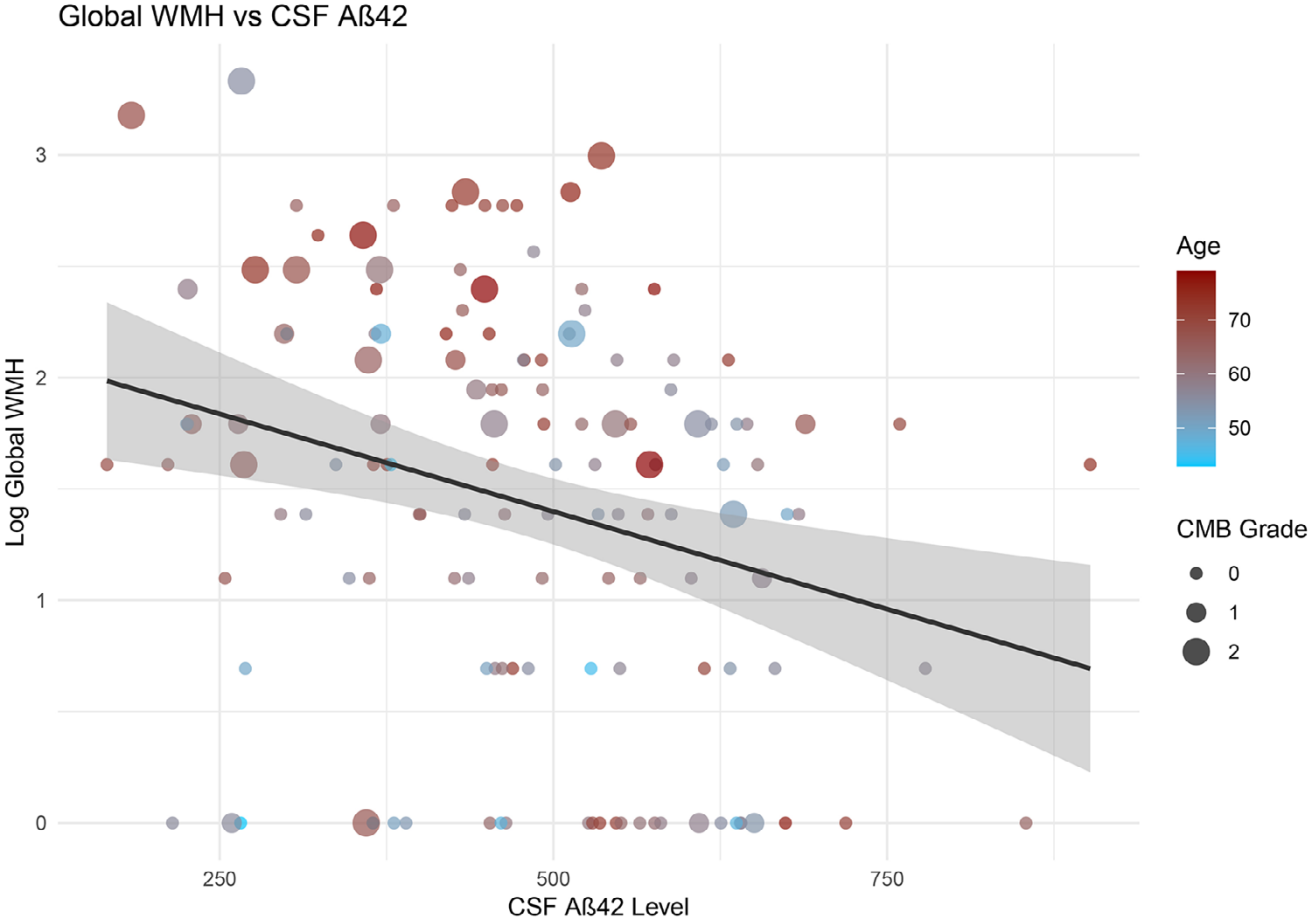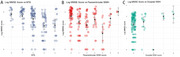# Global and Regional White Matter Hyperintensities in Alzheimer's Disease: Exploring Etiologies and Cognitive Correlates

**DOI:** 10.1002/alz70856_102956

**Published:** 2025-12-25

**Authors:** Yuyue Qiu, Jialu Bao, Tianyi Wang, Li Shang, Shanshan Chu, Wei Jin, Yuhan Jiang, Wenjun Wang, Bo Li, Yixuan Huang, Yunfan You, Yuanheng Li, Bo Hou, Longze Sha, Dongjing Li, Ling Qiu, Qi Xu, Feng Feng, Liling Dong, Chenhui Mao, Jing Gao

**Affiliations:** ^1^ Peking Union Medical College Hospital, Beijing, Beijing, China; ^2^ Institute of Basic Medical Sciences & Neuroscience Center, Chinese Academy of Medical Sciences and Peking Union Medical College, Beijing, Beijing, China

## Abstract

**Background:**

White matter hyperintensities (WMH) are commonly observed in the elderly, particularly in individuals with Alzheimer's disease (AD), but the underlying pathophysiology remains unclear. This cross‐sectional study aims to explore the etiologies and cognitive correlates of both global and regional WMH in a cohort of biologically diagnosed AD patients.

**Method:**

A cohort of 170 AD patients with cerebrospinal fluid (CSF) AD biomarkers, MRI scans, Clinical Dementia Rating (CDR), and Mini‐Mental State Examination (MMSE) scores, along with systemic vascular risk factor assessments, were included in this study. Atrophy and WMH were assessed by semiquantitative scales. Linear regression models were performed to evaluate the associations between global and regional WMH and factors such as age, cerebral microbleeds (CMB), AD biomarkers, cortical atrophy, and vascular risk scores. Additionally, linear regression analysis was performed to explore the relationship between MMSE scores and WMH and atrophy burden, adjusting for age, sex, and years of education.

**Result:**

Older age (β=0.03, SE=0.01, *p* < 0.001), lower Aβ42 level of CSF (β=‐0.0014, SE=0.0005, *p* = 0.01) and more severe CMB grade (β=0.48, SE=0.16, *p* = 0.003) were significantly associated with increasing global WMH score among AD patients. Analysis of regional WMH burden revealed significant associations with age (paraventricular: β=0.02, SE=0.01, *p* = 0.0003*, FDR.p=0.01*; frontal: β=0.01, SE=0.01, *p* = 0.03* FDR.p=0.27; parietal: β=0.02, SE=0.01, *p* = 0.00008*, FDR.p=0.01*), CMB grade (paraventricular: β=0.35, SE=0.11, *p* = 0.002*, FDR.p=0.05*; frontal: β=0.3, SE=0.11, *p* = 0.01*, FDR.p=0.09; parietal: β=0.33, SE=0.11, *p* = 0.004*, FDR.p=0.06), and CSF Aβ42 level (paraventricular: β=‐0.0008, SE=0.00035, *p* = 0.03*, FDR.p=0.27; frontal: β=‐0.0007, SE=0.00036, *p* = 0.04*, FDR.p=0.3; parietal: β=‐0.0009, SE=0.00035, *p* = 0.01*, FDR.p=0.1) in the paraventricular, frontal, and parietal lobes. Cognitive analysis revealed that after adjusting for age, sex, and years of education, decreased cognitive performance (MMSE score) was significantly associated with progressive medial temporal atrophy (MTA) (β=‐0.82, SE=0.23, *p* = 0.00056) and increased paraventricular WMH burden (β=‐0.22, SE=0.05, *p* = 0.00005).

**Conclusion:**

In AD patients, WMH—particularly in the frontal, parietal, and paraventricular regions—are associated with AD‐related pathology, including CSF Aβ42 levels and CMB, but not with CSF tau levels or vascular risk factors. Notably, paraventricular WMH are significantly linked to cognitive decline, independent of cortical atrophy.